# Short-term effects of foot surgery on walking-related pain, function, and satisfaction in patients with Charcot–Marie–Tooth disease: a prospective cohort study

**DOI:** 10.3389/fneur.2023.1304258

**Published:** 2024-01-10

**Authors:** Giacomo Basini, Chiara Rambelli, Martina Galletti, Paolo Zerbinati, Paolo Prati, Francesca Mascioli, Stefano Masiero, Davide Mazzoli, Andrea Merlo

**Affiliations:** ^1^Gait and Motion Analysis Laboratory, Sol et Salus Hospital, Torre Pedrera di Rimini, Italy; ^2^Department of Neuroscience, Section of Rehabilitation, University of Padova, Padova, Italy; ^3^Neuro-Orthopedic Unit, Sol et Salus Hospital, Torre Pedrera di Rimini, Italy

**Keywords:** Charcot–Marie–Tooth disease, pain, neuro-orthopedic surgery, rehabilitation, center of pressure, ankle range of motion

## Abstract

**Introduction:**

Patients with Charcot–Marie–Tooth disease (CMT) often suffer from walking-related pain (WRP), muscle weakness, foot deformities, and reduced ankle dorsiflexion (DF), which affects their ability to walk and daily activities. Functional surgery (FS) can restore foot deviations, affecting the loading ability during gait. We assessed the short-term effects of FS in patients with CMT on WRP, foot and ankle structure, and function, along with patients’ perceived improvement.

**Methods:**

This is a prospective cohort study on CMT patients who had undergone FS and rehabilitation. We analyzed the changes after 1 month, focusing on WRP, DF, the center of pressure progression (COPP) during walking, and measures of walking ability. The non-parametric Wilcoxon test was used.

**Results:**

Ten patients were included. One month after FS, WRP reduced from 5.5 (IQR = 3.5) to 2 (IQR = 3.5), *p* = 0.063, with an effect size of 0.615. The highest decrease was found in patients with very high pre-surgical pain levels. DF almost reached 10° for both active and passive movements (*p* < 0.05), and COPP improved from 44 to 60% (*p* = 0.009) of foot length. Gait speed, lower limb functioning, and balance did not change. More than half of the sample felt improved or much improved after FS.

**Conclusion:**

FS can be effective in reducing WRP and restoring foot posture in CMT patients in the short-term, which allows them to wear shoes, and leads to a perceived improvement and satisfaction. Lack of improvement in functional skills may be due to muscle weakness typical of CMT. Studies with longer follow-ups may confirm these hypotheses.

## 1 Introduction

Charcot–Marie-Tooth (CMT) disease is one of the most frequent inherited neuromuscular conditions, affecting nearly 4 out of 10,000 individuals (1, 2). It is a progressive peripheral motor and sensory neuropathy. CMT disease includes a variety of clinical and genetic forms very different from each other in terms of severity and prognosis (2). The first clinical signs to arise in CMT patients are progressive weakness and muscle atrophy of the foot and leg. Agonistic and antagonistic muscles are affected differently in terms of timing and severity, resulting in an imbalance between them and leading to cavo-varus foot deformities, claw-toes, and foot drops during walking (3). Other manifestations include sensory impairment, a reduction of tendon reflexes, and muscle cramps (2, 4). Foot deformities represent one of the first pathological manifestations in these patients, reducing walking ability and performance of daily activities, increasing postural instability and risk of falling, and eventually affecting quality of life (QoL) (3, 5–7). Walking-related pain (WRP), ambulatory limitations, and difficulty in finding suitable shoes to wear are the main issues related to foot deformity, as reported by patients (8–10).

Functional surgery (FS) is used to correct foot deviations in CMT patients by restoring ankle-foot biomechanics and muscular balance. Surgical procedures have been progressively improving since the late 1970s, based also on the surgeons’ personal preferences and backgrounds (3). FS aims at restoring the physiological tibiotalar and foot joint biomechanics and correcting foot rockers during gait as best as possible, improving walking safety (11, 12). FS usually includes soft tissue procedures such as customized tendon transfer, muscle-tendon lengthening interventions, and bone procedures such as metatarsal osteotomy (13). Previous studies on foot FS in CMT patients have reported a lower rate of additional surgeries and joint degeneration after years compared to those who underwent triple arthrodesis (14). Improvement in the push-off phase during walking (15), increased walking efficiency (3, 13), and reduced pain (13) were also highlighted. Since CMT patients have an impaired push-off phase, it is likely to infer that the rocker ability of the foot on the ground may increase following FS. During the stance phase of gait, the tibia rotates forward over the tibiotalar joint, and the origin of the ground reaction force (GRF) – i.e., the Center of Pressure (COP) – moves forward from heel to toe. The physiological COP pattern starts medially under the heel when it strikes the ground, gradually shifts forward and laterally during the stance phase, and finishes medially under the first toe before the swing phase. A correct COP progression (COPP) indicates progressive forward body displacement along the sagittal plane, as allowed by the three rockers that occur during stance (16, 17). COPP in the walking direction can be used as a synthetic measure of the quality of foot rockers (18). In gait analysis, force plates are commonly used to measure GFR and COP (17, 19–22).

In patients with CMT, the restoration of foot shape, ankle joint mobility, and loading ability should result in an improvement in terms of progression, stability, and WRP. This is of particular interest when considering current surgical techniques where tendon lengthening, tenotomies, and osteotomies are used to restore both the anatomy and mobility of the foot-ankle complex, in contrast to previous approaches based on joint arthrodesis (3). Such analysis is missing in the literature.

The aim of this study is to assess the short-term effects of FS to correct foot deformities on WRP, foot and ankle structure and function, and patient satisfaction in adult patients with CMT disease.

## 2 Methods

### 2.1 Design

A prospective cohort study.

### 2.2 Primary outcome

In this study, WRP was selected as the primary outcome. Patients were asked to specify whether any pain in the operated lower limb occurred during walking and where this was located. For patients who underwent bilateral surgery, a distinction was made for the two limbs. This was assessed by the Numerical Pain Rating Scale (NPRS). NPRS is a validated and globally used scale for pain assessment, with excellent metric characteristics. It is scored between 0 (no pain) and 10 (worst pain imaginable), with one-step increments (23). We considered a minimally clinically important difference (MCID) of two points (24).

### 2.3 Secondary outcomes

The secondary outcomes of this study involve structure and function domains, according to the International Classification of Functioning, Disability, and Health (ICF).

Structure-related outcomes were ankle passive and active dorsiflexion (aDF and pDF) assessed with the extended and flexed knee (KE and KF). Ankle DF was measured with a handheld goniometer with a fulcrum on the lateral malleolus and arms parallel to the tibia and the fifth metatarsal bone.

Regarding function-related outcomes, we measured the impact of the neuropathy on walking, as assessed by the Walk−12 scale (25).

Instrumental gait analysis was carried out by a nine-camera motion capture system (SMART-DX, BTS Bioengineering, Milan, Italy), four force platforms (Infinity P6000, BTS Bioengineering, Milan, Italy), and three video cameras. Reflective markers were placed according to the conventional protocol (26). Patients walked at a self-selected speed on a 12 m-long walkway. A minimum of three trials per subject was considered. Data were analyzed using the Propulsion Easy Report tool of the MADAM (Motion Analysis DAta Management software, Merlo Bioengineering, Italy). Walking speed was computed as the ratio between the forward displacement of a marker placed on the sacrum (within the calibration volume of approximately 4 meters in our laboratory) and the time required for completing this distance. The average value over the available trials (3–5) was computed and normalized by body height (27). Peak dorsiflexion during stance was computed from ankle kinematics to quantify the recovery in joint ROM during walking. The overall variation in the ankle kinematics profile was also visually inspected and discussed.

COPP in the walking direction was computed as the difference between the longitudinal COP coordinates at foot-off and heel strike. COPP was then normalized to foot length to obtain comparable and homogeneous data among the sample. In the computation procedure, a threshold value of 20 N to the vertical component of the GRF was set to identify the onset and the end of the stance phases. This allowed disregarding data at the very beginning and the very end of the stance phase (28) where the vertical force approaches 0 N and the estimate of COP position – computed as the ratio between the horizontal moments and the vertical force – becomes unreliable. GRF components and COP displacement were plotted and visually inspected for all subjects and trials. For all instrumental indices, the median value among trials was computed and used for further analyses.

The other function-related outcomes were balance assessed with the Berg Balance Scale (BBS) (29) and lower limb functioning assessed with the Short Physical Performance Battery (SPPB) (30).

Finally, the Patient Global Impression of Change (p-GIC) was used to assess patients’ perceptions related to changes after surgery. The scale goes from 1 (much improved) to 7 (much worsened) (31). All clinical assessments were performed by one experienced physiotherapist.

### 2.4 Setting

All assessments and interventions were delivered at the Sol et Salus Hospital in Rimini, Italy. Initial clinical and instrumental evaluations were scheduled at most 6 months before surgery.

The study protocol was approved by the local Ethics Committee (CEROM 2198/2018).

### 2.5 Patients

Patients were included in the study according to the following inclusion criteria: (1) age ≥ 18 years; (2) previous neurologic diagnosis of CMT disease (either genetic or based on clinical and electromyographic assessment); (3) referral for corrective foot surgery; (4) ability to walk barefoot for 10 meters, with or without aids; (5) available instrumental data both before and after surgery; (6) available signed informed consent for participating in the study.

Patients were excluded in cases of (1) severe cognitive impairments or inability to actively participate in post-surgery rehabilitation and follow instructions; (2) focal inhibition procedures on the lower limb muscles during the 6 months prior to evaluation; and (3) contraindications to physiotherapy and rehabilitation.

Recruitment started in February 2017 and lasted until January 2021. The selection of participants to be included in the study was carried out by a physical medicine and rehabilitation doctor (author CR).

### 2.6 Intervention

FS was performed by the same surgeon (author PZ). Some patients needed correction to both lower limbs, described in the following text as “bilateral surgery.” When this was performed, it took place in two different sessions, one for each limb, approximately a year apart. Tailored corrections were performed according to the initial clinical and instrumental assessment based on gait analysis and are listed in the Supplementary material (32).

In the case of soft tissue surgery only, rehabilitation began immediately after 2 days and was delivered as inpatients. Patients wore a non-articulated AFO orthosis to gradually increase weight bearing. When surgery also included major bone correction procedures (i.e., Akron dome osteotomy and percutaneous calcaneal valgus osteotomy), patients wore a cast for 1 month at home, with a recommendation to avoid limb loading, and then came back to inpatient care to begin rehabilitation after X-ray examination. All procedures were conducted as part of routine clinical care. The hospitalization lasted 21–28 days and patients received usual rehabilitation, including postural hygiene, a progressive stretching program, passive and active mobilization of the whole lower limb, and gait training. The physiotherapy program was designed according to the neuro-orthopedic surgeon’s recommendations. Rehabilitation was administered twice a day and six sessions per week up to 100–110 min each for 30 days. Patients were assessed before surgery and at the end of the rehabilitation period. When discharged, participants were instructed to continue the exercises, and a strengthening program was provided when authorized by the surgeon.

### 2.7 Statistical analysis

Descriptive statistics were used to summarize available data. The non-parametric Wilcoxon Test was used to verify the presence of improvement between the baseline and follow-up values in clinical and instrumental assessments.

The sample size was based on the number of patients treated at our Institution who satisfied the inclusion criteria during the study period.

## 3 Results

### 3.1 Participants

A total of 12 adult patients were screened, and 10 of them fulfilled the inclusion criteria. Post-surgical force plate data were not available for two subjects. All patients signed the informed consent to participate in the study. Two of the included patients underwent surgery for both lower limbs over two consecutive years. For this reason, all variables except demographics are presented twice, resulting in 12 analyzed lower limbs. Three patients underwent major bone surgery, including Akron Dome and percutaneous calcaneal valgus osteotomy. After surgery, they went home with a recommendation to avoid load-bearing and came back for rehabilitation after 1 month.

### 3.2 Descriptive data

Table 1 describes the sample characteristics, including demographics, CMT diagnosis details, surgical side, and use of walking aids and orthoses.

**Table 1:** Sample characteristics at baseline evaluation.

	Baseline values (*N* = 10)
Age	40.3 (13.7); 25–64
Gender (M/F)	5/5
CMT type	
CMT 1A	4
CMT X-linked	1
Not identified	5
Surgical side (R/L)^a^	6/6
Use of walking aids	0
Use of orthoses (insoles, knee pads)^a^	7

Data are reported as median (IQR) and range for continuous variables and as counts for nominal variables.

^a^Two patients underwent bilateral surgery, so side-specific variables are reported for both limbs.

### 3.3 Primary outcome

The variation of WRP, 1 month after surgery is presented in Table 2. WRP after surgery was localized mainly to the malleolar and tibiotalar regions, metatarsal bones, and triceps surae. When considering unilateral surgery, patients with a severely impairing pre-surgical WRP level reported a sizeable decrease in pain. Similar results were found for patients who underwent bilateral surgery, with no significant differences between groups (*p* = 0.731). Conversely, an increase of three points was reported by three of the patients with the lowest pre-surgical NPRS values. This increase was due to an ill-suited fit of the pre-surgical orthosis with the current leg and foot anatomy, as corrected by FS.

**Table 2:** One-month variation of walking-related pain as measured by the Numerical Pain Rating Scale, sorted in descending order according to the baseline value.

Pre-surgical NPRS	Post-surgical NPRS
10	1
8	1
7 ^a^	0
7 ^a^	4
7 ^b^	2
6	5
5	2
5	0
4	7
2 ^b^	5
1	0
0	3

NPRS, numerical pain rating scale; ^a^same subject; ^b^same subject. Two patients underwent bilateral surgery, so side-specific pain is reported for both limbs.

The median level of WRP in the sample decreased from 5.5 (IQR = 3.5, range 0–10) to 2 (IQR = 3.5, range 0–7, *p* = 0.063) with an effect size of 0.615. Noteworthy, both the 75th and the 90th percentiles were ≤ 5 at the one-month mark.

### 3.4 Secondary outcomes

Table 3 shows comparisons between pre- and post-operative values, computed using the Wilcoxon test. At baseline, equinus foot deformity was present in almost all sample patients, slightly reduced at passive mobilization. Patients walked at a slower speed, compared to the physiological reference of 75–85% height/s, and moderately complained of how the neuropathy impacted their walking.

**Table 3:** Comparison between pre- and post-operative values.

	Baseline	Post-intervention	value of *p*
Ankle dorsiflexion_KE^a^			
pDF_KE	-1° (6°); −15° – 5°	10° (6°); 0° – 20°	0.007
aDF_KE	-12° (8°); −25° – 0°	1° (6°); −10° – 10°	0.002
Ankle dorsiflexion_KF^a^			
pDF_KF	3° (8°); −15 – 15°	13° (7°); 0° – 20°	0.014
aDF_KF	-7° (8°); −25° – 0°	4° (6°); −5° – 10°	0.008
Walk-12 scale	39 (16); 19–51	31 (8); 23–48	0.066
Walking velocity (% height/s)	46 (16); 19–63	40 (10); 24–60	0.346
Peak dorsiflexion during stance	7° (8°); −10° – 16°	13° (4°); 6° – 18°	0.012
Center of pressure progression (% foot length) ^a^	44 (1); 33–66	60 (13); 29–72	0.009
Berg balance scale	47 (7); 30–54	49 (5); 36–53	0.136
Short physical performance battery	10 (3); 3–12	10 (3); 6–11	0.423
Patients’ global impression of change^a^	-	1.50 (1.25); 1–5	-

^a^Two patients underwent bilateral surgery, so side-specific variables are reported for both limbs.

Data are presented as mean (SD) and range for continuous variables, and as median (IQR) and range for ordinal variables.

**Figure 1:**
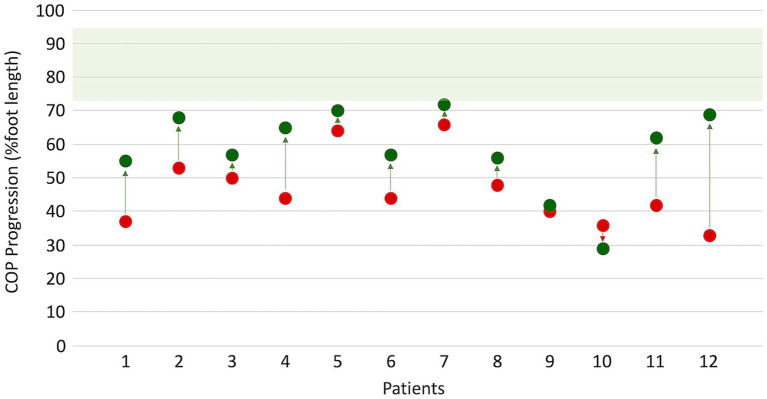
Changes in center of pressure progression (COPP) before (red dots) and after (green dots) surgery. Normative values (green band) were obtained from 12 age-matched healthy subjects walking at a matched slow speed of 47 (9) %height/s.

After surgery, a significant improvement in ankle ROM was found (*p* < 0.05), with almost 10° achieved toward dorsiflexion for both active and passive movements in both KE and KF conditions. Walking speed showed an insignificant decrease after surgery, as expected. Even in dynamic conditions, peak dorsiflexion during stance significantly increased by 6° in the sample (*p* = 0.012). At the visual inspection of ankle kinematics, eight patients showed improvement, mainly due to the restoration of the first and second ankle rockers. Conversely, ankle dorsiflexion during swing did not recover after FS. The Walk−12 score, which involves how the neuropathy impacts the ability to walk, decreased from 39 to 31, with a 6.6% probability that this improvement was due to chance. COPP improved from 44 to 60% (*p* = 0.009), indicating a recovery in both foot positioning and body-forward progression over the stance foot. As for WRP, the largest improvements were found for the most compromised patients (see Figure 1). A few patients reached values close to the lower value of the normality range for COPP.

Balance and lower limb physical functioning, assessed by the BBS and the SPPB, did not change.

Patients described themselves as “improved” or “much improved,” with a median p-GIC of 1.5 (range 1–5). When asked about the reasoning behind this assessment, most of them answered that they felt better because either the WRP subsided or they could use off-the-rack shoes after surgery.

## 4 Discussion

This study aimed to describe how WRP changes in the short term after FS and rehabilitation in patients with CMT and lower limb deformities. We also analyzed the evolution of indicators related to structure and function, according to the ICF model, along with patients’ perception of such changes.

We found a median decrease of three points at the NPRS, indicating a clinically meaningful improvement in pain during walking. The comparison at the Wilcoxon test was borderline significant, with a probability of 6.3% that the decrease was due to chance. The standard level of 5% of alpha error was not reached due to the WRP increase in 3 out of 12 patients, which was probably linked to the still-recent post-operative period and the previous orthoses not fitting properly with the new shape of the foot. It is, however, noteworthy to highlight that some patients experienced a decrease of more than six points (three times the MCID), going from 10 to 1, or from 8 to 1 in the pre- and post-operative conditions. A longer follow-up would have been necessary to evaluate the trend of this variable and eventually confirm the effect of time on healing. Pain, both neuropathic and related to foot deformity, is one of the main concerns of CMT patients. Pain-free walking is a factor that can strongly influence functional movements (33). The potential of FS and subsequent rehabilitation to reduce WRP in such a short time, even by several points beyond the MCID, is a promising aspect that deserves further investigation.

Abnormal foot posture can be associated with increased odds of falling and injury, thus affecting patients’ mobility, participation, and QoL in the long term (34). Moreover, foot deformities such as those usually found in CMT patients might prevent them from wearing shoes (35), which is one of the patients’ main concerns influencing both social life and self-image (36–38).

One month after surgery, all measurements of ankle DF significantly improved both at the bedside and during walking, thanks to FS and subsequent physiotherapy. The recovery of ankle ROM, especially during loading response and initial stance, contributed to promoting greater COPP, which increased from 44% of foot length to 60%. COPP measures the effect of the deformity on the quality of the foot-ground interface, the load-bearing capacity, and the progression of body weight on the foot during stance. The latter can be easily measured with pressure sensors and force platforms from motion analysis laboratories during walking (21). Restoring joint ROM allows for the recovery of the first and second rockers of the foot during gait, being essential prerequisites for functional walking (3, 34, 39). Patients who underwent foot FS are likely to cope more successfully with the loading and propulsion phases of gait (15). Therefore, an improvement in COPP may promote a stable and safe gait pattern.

Improvement in the ICF structure domain did not match up with enhanced function in our sample. Patients complained of moderate impact of their neuropathy on walking function, scoring 39/60 on the Walk-12 scale, which was reduced by approximately 8 points after surgery, and with a 6.6% probability, this result was due to chance. Physical performance at the SPPB remained stable after 1 month. Patients included in this study were just above the cut-off of fall risk without aids, according to the baseline score obtained at the BBS. One month after surgery, this slightly improved with no significant changes. On the one hand, our follow-up was probably too short to observe any improvement in the domain of function. On the other hand, distal muscles are usually weaker in this population (34), so patients may not be able to have the necessary strength to increase propulsion, gait speed, and functional mobility, even when foot deformities and soft tissue brakes are surgically reduced. Weakness and a short follow-up may be the main reasons we did not find any functional improvement in walking speed, lower limb function, and balance. The lack of active ankle dorsiflexion during swing aligns with dorsiflexor weakness due to peripheral neuropathy. Moreover, patients with CMT fashioned their gait over the years, with chronic deformities affecting the gait pattern. Corrective interventions such as those performed in this study may lengthen the amount of time it takes patients to adjust to the new limb structure and consolidate a new walking pattern that is effective and safe.

Overall, patient perception of having improved was high, as shown by the median value of 1.5 at the p-GIC, between “improved” and “much improved.” The satisfaction of patients’ expectations is key when performing surgery and setting up rehabilitation programs since surgery and rehabilitation should always be patient-centered and address the patients’ needs. This improvement is probably linked to a recovery in foot flexibility subsequent to surgery, which also results in partial recovery in the foot rockers allowing for the increase in COPP. Moreover, most of the patients stated that their satisfaction level and perception of improvement were linked to the possibility of wearing off-the-rack shoes or less cumbersome orthopedic ones. Other factors might also be at play, such as an improved self-image, a better social life, the possibility of walking with lighter orthoses or without orthopedic footwear, and less WRP when wearing shoes (36–38).

### 4.1 Limitations

This study has some limitations that should be addressed with proper deliberation.

The main drawback is a limited sample size, which is mainly due to the fact that CMT is a rare genetic disease with an incidence of four out of 10,000 individuals (1, 2). The choice of not including underage patients further reduced the sample. However, merging data of both children and older adults would have been misleading.

The absence of long-term data represents another important limitation of the study. Our institution performs FS on patients from all over the country. Long-term follow-up evaluations are purely based on clinical assessments and are performed by our surgeon at other facilities closer to the patients’ homes. Unfortunately, structured data collection is not available for these assessments. New studies should be designed to address the long-term effects of foot surgery in these patients.

## 5 Conclusion

In conclusion, this study described the short-term effects of FS and a subsequent physiotherapy program for the correction of lower limb deformities in patients with CMT. WRP decreased. Ankle dorsiflexion and COPP rose considerably in the short term, supporting the idea of potential improvements in other variables in the months to come.

## Data availability statement

The raw data supporting the conclusions of this article will be made available by the authors, without undue reservation.

## Ethics statement

This study was approved by the Comitato Etico della Romagna - CEROM (ID 2198/2018). The study was conducted in accordance with the local legislation and institutional requirements. The participants provided their written informed consent to participate in this study.

## Author contributions

GB: Writing – review & editing. CR: Conceptualization, Formal analysis, Methodology, Writing – original draft, Writing – review & editing. MG: Writing – review & editing. PZ: Writing – review & editing. PP: Data curation, Writing – review & editing. FM: Data curation, Writing – review & editing. SM: Writing – review & editing. DM: Conceptualization, Formal analysis, Methodology, Supervision, Writing – review & editing. AM: Conceptualization, Data curation, Formal analysis, Methodology, Writing – original draft, Writing – review & editing.
