# Supplementary material for: Short-term effects of foot surgery on walking-related pain, function, and satisfaction in patients with Charcot–Marie–Tooth disease: a prospective cohort study
Source: Front Neurol. 2024 Jan 10;14:1304258. doi: 10.3389/fneur.2023.1304258 (PMC10807423; doi:10.3389/fneur.2023.1304258)
Supplement: Supplementary file 1 [file Table_1.docx]

Supplementary Material

# Supplementary Table - Description of the surgical procedures undergone by the sample patients.

| **Surgical interventions** | **Number of surgeries** |
| --- | --- |
| **Bone surgery** |  |
| *First metatarsal percutaneous osteotomy* | 10 |
| *Akron-Dome osteotomy* | 2 |
| *Percutaneous calcaneal valgus osteotomy* | 1 |
| *Periastragalic artrotomy* | 1 |
| *Akin corrective osteotomy* | 1 |
| **Soft tissue surgery** |  |
| *Plantar fascia release* | 10 |
| *Peroneus longus tenodesis on peroneus brevis/ Peroneus longus tenotomy* | 11 |
| *Vulpius procedure/Achilles tendon percutaneous lengthening/Gastrocnemii lengthening/Hoke procedure/ Triceps surae fibrotomy* | 9 |
| *Flexor digitorum tenotomy/ Flexor hallucis longus lengthening/ Flexor digitorum longus lengthening* | 11 |
| *SPLATT* | 4 |
| *Second metatarsal extensor tenotomy* | 1 |
| *Tibialis posterior reinsertion* | 1 |
